# What Is the Draw of the Metaverse? Personality Correlates of Zepeto Use Motives and Their Associations With Psychological Well-Being

**DOI:** 10.1089/cyber.2022.0164

**Published:** 2023-03-06

**Authors:** Eun-Ju Lee, Wonjae Lee, Inhwan Bae

**Affiliations:** ^1^ Department of Communication, Seoul National University, Seoul, Republic of Korea.; ^2^ Graduate School of Culture Technology, Korea Advanced Institute of Science and Technology (KAIST), Daejeon, Republic of Korea.; ^3^ Institute of Communication Research, Seoul National University, Seoul, Republic of Korea.

**Keywords:** Big Five personality traits, loneliness, metaverse, social support, uses and gratifications, Zepeto

## Abstract

Despite the hype surrounding the metaverse, there is scant empirical research that examines who uses the service, for what specific purposes, and with what consequences. Based on a survey of current Zepeto users (*N* = 200), a popular metaverse application that enables people to create avatars and socialize while exploring the virtual spaces, we investigated (a) the key motives of Zepeto use, (b) how Big Five personality traits predict specific motives of Zepeto use, and (c) how specific motives of Zepeto use are associated with users' psychological well-being. Overall, users were largely driven by the desire to explore the virtual world and enjoy unique experiences, but such a tendency was stronger among those higher on openness and agreeableness. Extroverts were more likely to use Zepeto for functional purposes, while those higher on neuroticism turned to Zepeto to escape from reality. As for psychological consequences, while those using Zepeto for functional and escaping purposes reported higher levels of loneliness, those who used Zepeto for social and experiential goals were less lonely. The experiential and escape motives predicted perceived social support in the opposite directions. Moreover, by comparing Zepeto users' responses with those of non-users (*N* = 200), we found that (a) non-users overestimated users' motives of Zepeto use, especially social and escape motives, (b) Zepeto users were higher on extraversion and openness than non-users, and (c) users reported higher levels of loneliness than non-users with no significant difference in perceived social support. Implications of the findings and future directions are discussed.

## Introduction

Although there is no agreed-upon definition of what it is, there certainly exists all the hype about it—the metaverse.^1^ Heralded as the future of the internet or the internet of the future,^2^ the notion of metaverse has demanded much public attention, whether it takes the form of virtual meeting places (e.g., Gather), online games (e.g., Roblox), or a virtual social world where avatars meet and chat (e.g., Zepeto). While big technology companies like Microsoft and Meta are betting their future on the metaverse, less enthusiastic, if not entirely pessimistic, prospects co-exist, often referring to the apparent failure of its earlier forms, such as Second Life.

No matter how we predict the future trajectory of the metaverse, the COVID-19 pandemic has certainly accelerated its adoption. With significant restrictions on face-to-face interactions during the lockdown, people turned to various metaverse platforms, such as Zepeto, a popular metaverse application that has attracted over 300 million users worldwide since its launch in August 2018.^3^ Despite its fast-growing popularity, however, little is known as to how people understand the service, what specific motives drive its usage, what dispositional traits predict such motives, and what social psychological consequences follow its usage. In particular, how does the use of a social metaverse platform affect users' mental well-being, which has significantly worsened worldwide amid the global health crisis?^4^ Using an online survey of Zepeto users and non-users, this research addresses these questions.

## The Current Study

According to the use and gratification model, individuals choose to use media (among other alternatives) as a means to fulfill their unmet needs.^5^ Research on social virtual world identified various motives of use, such as friendship, escapism, role-playing, achievement, relationship, and manipulation.^6^ Based on an open-ended survey, Zhou et al^7^ extracted three types of needs that drove Second Life use: utilitarian needs (e.g., learning, shopping, creating, making money), hedonic needs (e.g., exploring, diversion, vicarious experience, escapism), and social needs (e.g., socializing, romance), which were later referred to as functional values, experiential values, and social values, respectively.^8^

While uses and gratifications research focuses mostly on the current users' motivations of media use and its consequences, for an emerging service like the metaverse, it is important to understand how non-users conceive of the service. By examining lay people's beliefs about what primary functions the metaverse service can fulfill (e.g., folk theories),^9^ we can better predict users' behaviors and experiences in the virtual world, and moreover, design more effective and gratifying services by enhancing the task-media fit.^10^ To that end, we (a) measured both Zepeto users' own motives of use and their perceptions of other users' motives and (b) compared Zepeto users' responses with those of non-users.

RQ1a-b. (a) What are the primary motives of Zepeto use? (b) Why do users and non-users think others use Zepeto?

At the same time, a host of individual differences shape individuals' specific media use as well as the motives underlying it. Most notably, although Big Five personality traits (openness, conscientiousness, extraversion, agreeableness, neuroticism) were not associated with the overall frequency of social network site (SNS) use,^11^ they tend to predict specific SNS behaviors. According to a meta-analysis, extraversion was related to more interaction on SNS and more SNS friends, while openness predicted information seeking, playing games, and status updates.^12^ In terms of motives of use, those higher on agreeableness were more likely to use Facebook to connect with and be accepted by others, but less likely to seek attention.^13^

Significant associations were also found between personality traits and motives of game play, such that while both extraversion and agreeableness were positively related to adventure, escapism, and achievement motives, it was only agreeableness that predicted the relationship motive.^14^ In a recent study, neuroticism positively predicted escapism and fantasy motives of game play, whereas the other four traits had inverse relationships with those motives.^14^ Considering that Zepeto is often equated with games, offering various features that cater to users' diverse needs, we explored (a) how personality traits as relatively stable individual differences lead people to seek different gratifications in the metaverse and (b) if certain personality traits differentiate between Zepeto users and non-users.

RQ2a-b: (a) How do users' Big Five personality traits predict their motives of Zepeto use? (b) Do Zepeto users and non-users exhibit different personality traits?

Among possible consequences of metaverse use, we focused on psychological well-being, which has become a critical issue around the globe amid the COVID-19 pandemic.^15^ In a Pew Research Center's survey in February 2021,^16^ 21 percent of U.S. adults reported high levels of psychological distress, a composite measure of anxiety, sleeplessness, depression, loneliness, and physical symptoms of distress. In particular, young adults 18 to 29 years of age were more likely to report anxiety, depression, or loneliness than those 30 years of age and older (45 percent vs. 28 percent). Similarly, a national survey in South Korea found a significant increase in those indicating high levels of depression from 3.2 percent in 2019 to 18.1 percent in 2021.^17^ In particular, we examined loneliness and perceived social support, which are directly associated with social interaction.

Given that restrictive COVID-19 measures such as social distancing and lockdowns have deprived individuals of the heretofore taken-for-granted opportunities to interact and socialize and that social isolation is a common cause of loneliness,^18^ it is no surprise that overall levels of loneliness heightened during the pandemic. With face-to-face interpersonal encounter being severely limited, those who used Zepeto especially for social interaction while navigating the virtual world, might have experienced less loneliness and felt increased social support. However, those who spend as much time in the metaverse for different purposes, for instance, to buy and sell virtual goods or to escape from real life problems, might not have experienced equivalent changes.

Given mixed findings concerning how online social interactions, especially those using social media, affect users' psychological well-being (see a recent comprehensive review^19^ and a meta-analysis^20^), however, we proposed the following research questions to elucidate the potential benefits of metaverse experiences (or the lack thereof):

RQ3a-b: Do Zepeto users and non-users report different levels of (a) loneliness and (b) perceived social support?

RQ4a-b: How are the motives of Zepeto use associated with the users' levels of (a) loneliness and (b) perceived social support?

## Methods

### Participants

Two hundred Zepeto users (65 men and 135 women, *M*_age_ = 22.82, *SD* = 6.59) and 200 non-users (65 men and 135 women, *M*_age_ = 22.77, *SD* = 6.10) were recruited by a survey company in South Korea. Considering that the 6- to 24-year-old cohort (Gen Z) accounts for more than 80 percent of Zepeto users,^21^ the sample consisted of 50 percent in their teens, 35 percent in their 20s, and 15 percent in their 30s for users and non-users, respectively. Gender imbalance also reflected the reported dominance of female users.^22^ This study was approved by the IRB at Korea Advanced Institute of Science and Technology.

### Measures

All items were measured using a 7-point scale (1 = *Strongly disagree* and 7 = *Strongly agree*), unless noted otherwise. While adopting items from Zhou et al^8^ (functional, social, and experiential values), we distinguished between experiential and escape motives, for each represents conceptually different gratifications sought and removing the escape motive item slightly heightened the reliability score of the experiential motive. Moreover, the motive measures demonstrated acceptable convergent validity (composite reliability >0.73 and average variance extracted >0.45; Table 1)^23^ and discriminant validity (heterotrait-monotrait ratio of correlations <0.64; Table 2).^24^

**Table 1.** Zepeto Use Motives

“I use Zepeto to…”
Functional motive (CR = 0.73, AVE = 0.48, α = 0.71, *M* = 3.37, *SD* = 1.42) (a) Shop/purchase goods, (b) sell virtual goods, (c) create objects/design virtual items
Social motive (CR = 0.89, AVE = 0.72, α = 0.88, *M* = 3.70, *SD* = 1.54) (a) Interact with people from all around the world, (b) maintain relationships with people that they already know, (c) build communities with similar others
Experiential motive (CR = 0.78, AVE = 0.46, α = 0.77, *M* = 4.90, *SD* = 1.18) (a) Explore the virtual world, (b) have fun, (c) pass time/overcome boredom, (d) engage in new experiences that cannot be gained in the real world
Escape motive (*M* = 3.40, *SD* = 1.62) (a) Escape from real-life problems and pressures

*N* = 200.

AVE, average variance extracted; CR, composite reliability; *SD*, standard deviation.

**Table 2.** Discriminant Validity of Measures of Zepeto Use Motives

	Functional motive	Social motive	Experiential motive
Social motive	0.63		
Experiential motive	0.50	0.63	
Escape motive	0.41	0.59	0.43

*N* = 200. Cell entries are HTMT values.

HTMT, heterotrait-monotrait ratio of correlations.

For perceived motives of (other) users, participants were presented with the same list and asked why they thought (other) users would use Zepeto. For non-users who might not be familiar with the Zepeto service, a short description of Zepeto was presented and they could choose “Don't know.”

Big Five personality traits were captured by “extraverted, enthusiastic” (extraversion: *M* = 3.69, *SD* = 1.81), “sympathetic, warm” (agreeableness: *M* = 4.87, *SD* = 1.36), “dependable, self-disciplined” (conscientiousness: *M* = 4.11, *SD* = 1.51), “anxious, easily upset” (neuroticism: *M* = 3.97, *SD* = 1.64), and “open to new experiences, complex” (openness: *M* = 4.69, *SD* = 1.45).^25^

For loneliness, participants responded to six items asking how often they felt connected with people around them (reverse-coded) and left out from others (e.g., “I feel alone”; 1 = *Never*, 7 = *All the time*; α = 0.85, *M* = 2.85, *SD* = 1.09).^26^ Perceived social support was measured by eight items tapping how supportive they thought their family and friends were, including “I get the emotional help and support I need from my family” and “I can count on my friends when things go wrong” (α = 0.92, *M* = 5.13, *SD* = 1.21).^27^

Users were also asked how often they accessed the Zepeto application last month (1 = *Never* and 9 = *Several times an hour*; *M* = 3.37, *SD* = 2.02) and how much time per day they spent using Zepeto (weekdays: *M* = 67.95 minutes, *SD* = 65.02, weekends: *M* = 105.25 minutes, *SD* = 89.07; overall *M* = 1.31 hours, *SD* = 1.10).

## Results

**FIG. 1.**
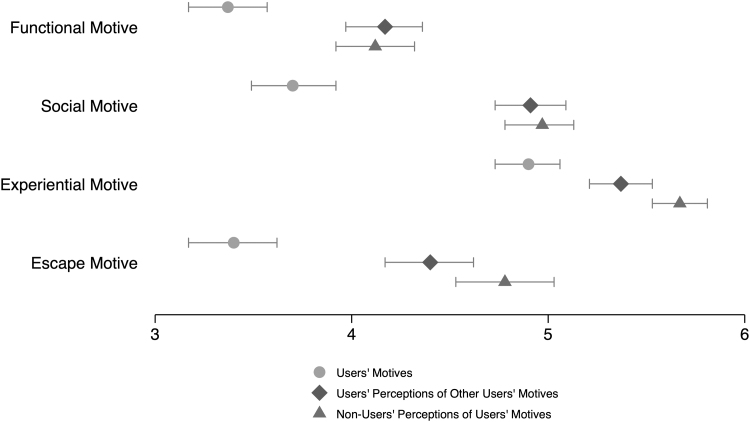
Means and 95 percent confidence intervals of users' motives, non-users' perceptions of users' motives, and users' perceptions of other users' motives.

*RQ1a* concerns the primary motives of Zepeto use. Zepeto users were most likely to use the service for experiential purposes, with the social motive being the distant second, followed by the escape and functional motives (Table 3 and Fig. 1). Paired-sample *t* tests confirmed that, except for the difference between the functional and escape motives, *t*(199) = −0.20, *p* = 0.840, the differences between any two motives were statistically significant, all *t*s > 2.90, *p*s < 0.005.

**Table 3.** Zepeto Users' Motives, Users' Perceptions of Other Users' Motives, and Non-Users' Perceptions of Users' Motives

Zepeto use motives	N	M	SD	t value for the equality to	
				Users' perceptions of other users' motive	Non-users' perceptions of users' motive
Functional					
Users' motive	200	3.37	1.42	−8.62^***^	−5.20^***^
Users' perceptions of other users' motive	200	4.17	1.37		0.32
Non-users' perceptions of users' motive	189^a^	4.12	1.42		
Social					
Users' motive	200	3.70	1.54	−11.65^***^	−9.10^***^
Users' perceptions of other users' motive	200	4.91	1.30		−0.41
Non-users' perceptions of users' motive	196^a^	4.97	1.19		
Experiential					
Users' motive	200	4.90	1.18	−7.81^***^	−6.98^***^
Users' perceptions of other users' motive	200	5.37	1.13		−2.77^**^
Non-users' perceptions of users' motive	198^a^	5.67	1.00		
Escape					
Users' motive	200	3.40	1.62	−8.49^***^	−8.09^***^
Users' perceptions of other users' motive	200	4.40	1.59		−2.28^*^
Non-users' perceptions of users' motive	178^a^	4.78	1.70		

^*^ *p* < 0.05; ^**^p < 0.01; ^***^p < 0.001.

^a^ “Don't know” responses were treated as missing.

To address why Zepeto users and non-users think other people use the service (*RQ1b*), independent-samples *t* tests and paired-sample *t* tests were conducted (Table 3 and Fig. 1). First, there was no significant difference between Zepeto users' and non-users' perceptions of how strong others' functional and social motives are. For the experiential and escape motives, however, non-users estimated them higher than users. Next, we compared Zepeto users' own motives with non-users' perceptions of users' motives to gauge how accurately non-users conjecture the actual users' motives. For all four Zepeto use motives, non-users overestimated the actual users' motives. Finally, Zepeto users' own motives and their perceptions of other users' motives were compared. Without exception, they perceived other users to have stronger motives than they themselves did.

*RQ2b* asks if Zepeto users and non-users differ in personality traits. As shown in Table 4, Zepeto users were more extraverted and open to experience. Likewise, we examined whether Zepeto users and non-users experienced different degrees of loneliness (*RQ3a*) and social support (*RQ3b*). Zepeto users were more lonely compared to non-users, but perceived no less social support (Table 4).

**Table 4.** Personality Traits, Loneliness, and Perceived Social Support of Zepeto Users and Non-Users

	Users,* M *(*SD*)	Non-users,* M *(*SD*)	t(398)
Extraversion	3.93 (1.78)	3.45 (1.80)	2.68^**^
Agreeableness	4.93 (1.27)	4.81 (1.45)	0.88
Conscientiousness	4.19 (1.50)	4.03 (1.52)	1.06
Neuroticism	4.09 (1.58)	3.86 (1.70)	1.44
Openness	4.93 (1.40)	4.45 (1.47)	3.38^***^
Loneliness	3.02 (1.13)	2.68 (1.03)	3.14^**^
Perceived social support	5.06 (1.19)	5.20 (1.22)	−1.18

*N* = 400.

^**^ *p* < 0.01; ^***^p < 0.001.

To assess the relations among Zepeto users' personality traits, their motives of Zepeto use (*RQ2a*), and mental well-being (loneliness and perceived social support) (*RQ4a-b*), controlling for sex, age, and Zepeto usage (frequency and amount), we fitted a structural equation model with maximum likelihood estimation. Specifically, we estimated a just-identified model, generating a perfect fit to the data, χ^2^(0) = 0, comparative fit index = 1, root mean square error of approximation = 0, and standardized root mean squared residual = 0 (Table 5).

**Table 5.** The Effects of Personality Traits on Zepeto Use Motives, Loneliness, and Perceived Social Support

	Functional motive	Social motive	Experiential motive	Escape motive	Loneliness	Perceived social support
Extraversion	0.148^*^ (0.068)	0.135 (0.072)	−0.032 (0.054)	−0.053 (0.077)	−0.133^**^ (0.046)	0.198^***^ (0.051)
Agreeableness	0.044 (0.084)	0.222^*^ (0.090)	0.267^***^ (0.067)	0.029 (0.095)	−0.055 (0.058)	0.246^***^ (0.063)
Conscientiousness	−0.052 (0.081)	0.036 (0.086)	0.024 (0.064)	0.063 (0.091)	−0.048 (0.053)	0.014 (0.058)
Neuroticism	−0.127^*^ (0.064)	−0.0003 (0.068)	−0.041 (0.051)	0.223^**^ (0.072)	0.171^***^ (0.045)	−0.096 (0.049)
Openness	−0.077 (0.081)	−0.010 (0.086)	0.133^*^ (0.065)	0.145 (0.091)	−0.067 (0.054)	−0.012 (0.060)
Functional motive					0.149^**^ (0.054)	−0.052 (0.060)
Social motive					−0.133^*^ (0.060)	0.091 (0.066)
Experiential motive					−0.270^***^ (0.071)	0.201^*^ (0.078)
Escape motive					0.218^***^ (0.050)	−0.112^*^ (0.056)
Use frequency					−0.019 (0.042)	−0.002 (0.046)
Use amount					0.038 (0.063)	−0.120 (0.069)
Male	0.334 (0.212)	0.370 (0.225)	−0.159 (0.169)	0.592^*^ (0.238)	0.098 (0.143)	0.101 (0.157)
Age	0.026 (0.015)	0.033^*^ (0.016)	0.024^*^ (0.012)	0.035^*^ (0.017)	0.015 (0.011)	−0.0001 (0.012)
*R* ^2^	0.076	0.119	0.161	0.105	0.393	0.334

*N* = 200. Cell entries are unstandardized regression coefficients and standard errors in parentheses obtained from structural equation modeling with maximum likelihood estimation. The residual correlations among the four motives and those between the two mental well-being variables, as well as the correlations among the predictors were also estimated, but not shown here for brevity.

^*^ *p* < 0.05; ^**^p < 0.01; ^***^p < 0.001.

For *RQ2a,* while extraversion only predicted the functional motive, agreeableness was positively associated with both social and experiential motives. Neuroticism positively predicted the escape motive, whereas it had a negative association with the functional motive. The higher the openness, the stronger the experiential motive, but conscientiousness had no significant relationship with any of the motives.

The functional and escape motives positively predicted loneliness, whereas the social and experiential motives had negative associations with loneliness (*RQ4a*). For perceived social support, the experiential motive was a positive predictor, whereas the escape motive was a negative one (*RQ4b*).

**FIG. 2.**
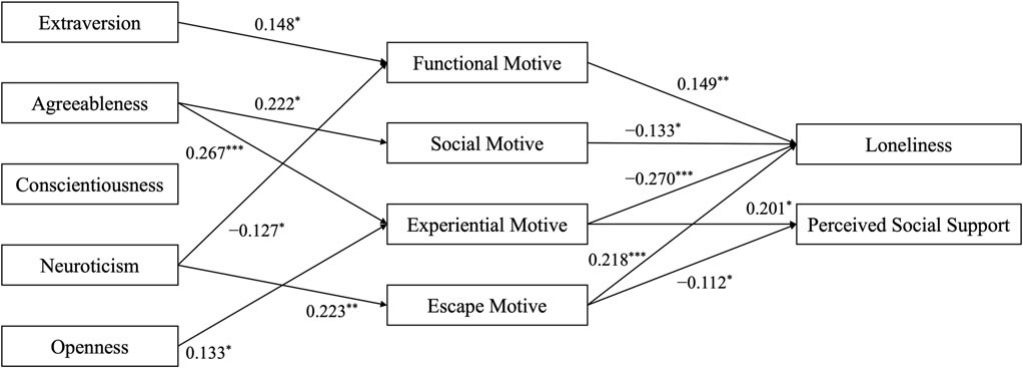
Path diagram of Zepeto users' personality traits, Zepeto use motives, and mental well-being. *N* = 200. *Values* denote unstandardized regression coefficients obtained from the structural equation model reported in Table 5. Only statistically significant paths are shown here for brevity. *p < 0.05; **p < 0.01; ***p < 0.001.

Tests of indirect effects of personality traits through Zepeto use motives revealed that extraversion increased loneliness by heightening the functional motive of Zepeto use (Table 6 and Fig. 2). By contrast, agreeableness, neuroticism, and openness reduced loneliness by either increasing the social (agreeableness) and experiential (agreeableness and openness) motives or decreasing the functional (neuroticism) motive. Neuroticism also had a positive indirect effect on loneliness by elevating the escape motive. Both agreeableness and openness increased perceived social support through the experiential motive, whereas neuroticism decreased it by fostering the escape motive.

**Table 6.** Indirect Effects of Personality Traits on Psychological Well-Being by Zepeto Use Motives

Independent variable (personality traits)	Mediator (use motives)	Dependent variable (mental well-being)	Indirect effect
Extraversion	Functional	Loneliness	0.022 [0.003 to 0.061]
Agreeableness	Social	Loneliness	−0.030 [−0.078 to −0.002]
	Experiential	Loneliness	−0.072 [−0.148 to −0.020]
	Experiential	Social support	0.054 [0.007 to 0.127]
Neuroticism	Functional	Loneliness	−0.019 [−0.058 to −0.001]
	Escape	Loneliness	0.049 [0.015 to 0.100]
	Escape	Social support	−0.025 [−0.069 to −0.003]
Openness	Experiential	Loneliness	−0.036 [−0.087 to −0.005]
	Experiential	Social support	0.027 [0.002 to 0.078]

*N* = 200. Cell entries are unstandardized indirect-effect coefficients and 95 percent bias-corrected bootstrap confidence intervals (based on 5,000 bootstrap samples) in brackets.

## Discussion

This research addresses emerging questions related to the metaverse, such as why people are attracted to the metaverse, what personality traits shape their motives of use, and how their psychological well-being varies as a function of those motives, by focusing on a specific metaverse platform, Zepeto. In so doing, we compared Zepeto users and non-users to see if there are distinct dispositional attributes that characterize Zepeto users.

Overall, Zepeto users turned to the service mostly to explore the virtual world, have fun, and engage in new experiences (i.e., experiential motive). Although the platform boasts itself as “an ultimate social platform,” meeting people from all around the world and maintaining existing relationships was a distant secondary motive. Indeed, when non-users' perceptions of why people use Zepeto were compared against the actual users' motives, non-users overestimated the strength of all four motives, but the discrepancy was particularly evident for the social and escape motives. Such findings suggest a gap between the public's perception of the service and the actual users' experiences.

Zepeto users were more extraverted and open to experience than were non-users, which is not surprising given the early adoption stage of the service. More intriguing is how each personality trait predicts specific motives of Zepeto use. Notably, while those more agreeable and open to experience expressed a stronger desire to seek novel experiences and have fun in the virtual world, those higher on neuroticism were more prone to escape from reality into the metaverse. Extraverted users turned to Zepeto for business purposes, but consciousness had no significant association with any of the motives. Moreover, experiential and escape motives each predicted mental health outcomes in the opposite directions. Although actively engaging in the unique offerings of the metaverse lowered loneliness while enhancing perceived social support, passively retreating into the cyberspace to avoid real-life issues worsened the user's psychological well-being, suggesting that these two motives should be distinguished.

Previous research yielded mixed findings as to how social media use predicts loneliness.^15^ In this study, while those driven by stronger functional and escape motives exhibited higher levels of loneliness, those who used Zepeto for social and experiential purposes were less lonely. Neither the frequency nor the amount of Zepeto use was significantly related to loneliness. Although we cannot unambiguously determine the causal direction using our cross-sectional data, it is not the sheer amount of metaverse usage, but specific motives of metaverse use that account for the changes in loneliness. Taken together with the finding that Zepeto users were lonelier than non-users, it seems reasonable to conclude that although lonely people are more attracted to the metaverse service, it is what they actively seek within that space that determines the psychological consequences they experience as a result.

On the other hand, the significant associations between Zepeto use motives and perceived social support were found only for the experiential and escape motives. This might have to do with the way perceived social support was operationalized. Using standardized scales, we asked about the participants' relationships with friends and family. As such, unless they had interacted with their friends and family in Zepeto, Zepeto use was unlikely to alter the amount of support they receive from existing ties. By expanding the boundary of social support network, future research should attempt to fully capture potential effects of metaverse use on mental well-being. Again, it merits note that those frequenting Zepeto to explore the novel experiences reported an *increase* in social support, whereas those using Zepeto to escape from reality indicated *reduced* social support.

Some limitations of this research deserve note. First, drawing upon the previous research on the virtual social world,^7,8^ we provided a predefined set of motives of Zepeto use, thereby foregoing an opportunity to identify newly emerging motives unique to the metaverse. It would be interesting to explore what gratifications are sought and obtained in the metaverse in an unrestricted manner, and furthermore, how they vary as a function of demographic variables, dispositional traits, and social and psychological needs. Relatedly, measuring the escape motive with a single item was less than desired. Although the escape motive is fairly unambiguous and narrow in scope, and thus relatively well-suited for a single-item measure, its reliability should be validated using multiple items.^28^

Second, due to the inherent challenges of defining the Zepeto user population, we relied on a convenience sample. Although this is a common issue when researching the users of specific media platforms and services, more efforts should be expended to comprise a representative sample that adequately reflects key characteristics of the actual user base.

Third, our results are based on cross-sectional data, and thus subject to alternative interpretations. To disambiguate the causal directions between motives of Zepeto use and psychological well-being, for instance, a longitudinal study is called for that addresses how individuals' metaverse experiences alter their psychological state over time, preferably beyond loneliness and perceived social support.

Fourth, we recruited only Korean users. Especially considering that the metaverse has no geographical boundary, it seems worthwhile to explore if there exists any cross-national or cross-cultural differences in why and how people use the service. Likewise, the metaverse is an umbrella term that encompasses a wide range of services with distinct technological and social affordances. Therefore, future research should examine if these findings replicate different metaverse services.

Our findings once again support the key premises of the uses and gratifications theory such that (a) personality traits shaped the specific motives of Zepeto use, which in turn, (b) affected the gratifications users obtain, while highlighting the conditional mental health benefits of Zepeto use during the COVID-19 pandemic. With the increasingly seamless integration of the virtual into our daily lives, the questions examined herein will certainly be of greater significance down the road. Who is more likely to use it, for what specific purposes, and with what consequences?
